# Yeast Biodiversity from DOQ Priorat Uninoculated Fermentations

**DOI:** 10.3389/fmicb.2016.00930

**Published:** 2016-06-15

**Authors:** Beatriz Padilla, David García-Fernández, Beatriz González, Iara Izidoro, Braulio Esteve-Zarzoso, Gemma Beltran, Albert Mas

**Affiliations:** Departament de Bioquímica i Biotecnologia, Facultat d’ Enologia, Universitat Rovira i VirgiliTarragona, Spain

**Keywords:** wine, Grenache, Carignan, *Saccharomyces cerevisiae*, Candida

## Abstract

Climate, soil, and grape varieties are the primary characteristics of *terroir* and lead to the definition of various appellations of origin. However, the microbiota associated with grapes are also affected by these conditions and can leave a footprint in a wine that will be part of the characteristics of *terroir*. Thus, a description of the yeast microbiota within a vineyard is of interest not only to provide a better understanding of the winemaking process, but also to understand the source of microorganisms that maintain a microbial footprint in wine from the examined vineyard. In this study, two typical grape varieties, Grenache and Carignan, have been sampled from four different vineyards in the DOQ Priorat winegrowing region. Afterward, eight spontaneous alcoholic fermentations containing only grapes from one sampling point and of one variety were conducted at laboratory scale. The fermentation kinetics and yeast population dynamics within each fermentation experiment were evaluated. Yeast identification was performed by RFLP-PCR of the 5.8S-ITS region and by sequencing D1/D2 of the 26S rRNA gene of the isolates. The fermentation kinetics did not indicate clear differences between the two varieties of grapes or among vineyards. Approximately 1,400 isolates were identified, exhibiting high species richness in some fermentations. Of all the isolates studied, approximately 60% belong to the genus *Hanseniaspora*, 16% to *Saccharomyces*, and 11% to *Candida*. Other minor genera, such as *Hansenula*, *Issatchenkia*, *Kluyveromyces*, *Saccharomycodes*, and *Zygosaccharomyces*, were also found. The distribution of the identified yeast throughout the fermentation process was studied, and *Saccharomyces cerevisiae* was found to be present mainly at the end of the fermentation process, while *Aureobasidium pullulans* was isolated primarily during the first days of fermentation in three of the eight spontaneous fermentations. This work highlights the complexity and diversity of the vineyard ecosystem, which contains yeasts from different species. The description of this yeast diversity will lead to the selection of native microbiota that can be used to produce quality wines with the characteristics of the Priorat.

## Introduction

Wine producers have recently grown concerns about the importance of introducing high quality wines to the market that exhibit geographical characteristics and complexity (Harvey et al., 2014). *Terroir* has been defined as the concept that links the sensory features of wine to the environmental conditions of vineyards. Climate, soil, and grape variety, among other factors, represent the main characteristics of a *terroir* (Van Leeuwen and Seguin, 2006). Moreover, these elements may condition what has been defined as the microbial biogeography of grapes (Bokulich et al., 2014), as unique microbial strains have been associated with specific geographical locations (Tofalo et al., 2013).

Different microorganisms, and particularly yeasts, are involved and play a key role in the production of wine. Grapes represent one of the main sources of the yeast populations found in wine (Mortimer and Polsinelli, 1999) and contain mainly non-*Saccharomyces* species; however, these species are gradually replaced by *Saccharomyces cerevisiae* throughout the process of alcoholic fermentation (Fleet, 1993). Recently, several non-*Saccharomyces* species have been related to positive attributes such as the production of interesting aroma compounds or the reduction of the final ethanol content of wine (Gonzalez et al., 2013; Jolly et al., 2014). Moreover, it has been reported that these species reach populations of up to 10^8^ CFU/mL during the alcoholic fermentation of wines (Combina et al., 2005). Therefore, the presence of non-*Saccharomyces* yeasts during vinification is likely to affect physico-chemical characteristics, leaving behind identifiable characteristics in the resulting wine.

To obtain wines that reflect a certain *terroir*, it is essential to reproduce industrially the microbial fingerprint of the spontaneous fermentations that occur during vinification while avoiding the microbiological and technological risks associated with uncontrolled fermentations. In this sense, the use of native yeasts is a feasible option (Carrascosa et al., 2012; Scacco et al., 2012), but the prior step of isolating and characterizing multiple yeast strains is essential to properly select strains (Tofalo et al., 2013). For this reason, ecological studies of vineyard yeast microbiota are of interest not only to better understand the winemaking process but also to determine the source of microorganisms that produce a particular microbial footprint. Many ecological studies of indigenous yeast microbiota from different vineyards have been published, and have been recently reviewed by Barata et al. (2012). Additional studies complement this information with microbial analyses of spontaneous alcoholic fermentations occurring in different winemaking regions (Torija et al., 2001; Combina et al., 2005; Díaz et al., 2013).

The Priorat Qualified Appellation of Origin (DOQ in Catalan) is a traditional area of wine production located in the south of Catalonia, Spain, where Carignan (CA) and Grenache (GR) are typical and characteristic red grape varieties. Although limited data exist concerning the microbial biogeography of grapes in DOQ Priorat, Torija et al. (2001) studied the yeast population dynamics of GR spontaneous fermentations in a cellar from Priorat, and determined that *Candida stellata* was the primary dominant non-*Saccharomyces* species. However, as different vineyards may broaden the microbial biodiversity of the region, the yeast population should be studied at different geographical points, and its dynamics should be observed under spontaneous conditions.

The aim of this study is to provide a detailed inventory of the yeast populations on GR and CA grapes and that could be developed in oenological conditions from DOQ Priorat. Berries from both varieties were collected at four different vineyards upon the ripening of the 2012 vintage, and spontaneous alcoholic fermentations were performed to characterize yeast population dynamics through the isolation and molecular identification of the yeasts present.

## Materials and Methods

### Grape Sampling and Spontaneous Fermentation

Four different vineyards (V1, V2, V3, and V4) were selected for the collection of both GR and CA grapes. All wine terraces belong to the Priorat DOQ and are between 300 and 800 m above sea level. In the Priorat region, most of the vineyards follow integrated approaches that attempt to minimize the use of pesticides and other chemicals. For each variety and each vineyard 2 kg of grapes were manually collected during vintage 2012 and transported refrigerated into sterile bags to the laboratory.

Grape juice was obtained after sterile manual selection, destemming and squeeze of 1.8 kg of berries. Must was placed at once with seeds and skin into 2 L sterile flasks. The spontaneous fermentation was conducted under agitation at 120 rpm and 24°C. The must was pumped up each 24 h, and after the first day 30 ppm of sulfur dioxide were added as potassium metabisulfite. Daily samples were withdrawn to monitor sugar concentration by measuring must density using an electronic densitometer (Mettler-Toledo S.A.E., Barcelona, Spain). In addition, samples of the grape juice (Day 0), before the addition of SO_2_ (Day 1), 24 h after the addition SO_2_ (Day 2) at a mid-fermentation point (M; density 1040–1060 g/L) and at the end of the fermentation (F) (density < 1000 g/L) were also aseptically taken for yeast counting and isolation.

### Yeast Content and Isolation

Aliquots of different serial decimal dilutions of samples were spread in duplicate on solid YPD (glucose 2%, peptone 0.5%, yeast extract 0.5%, and agar 2%) and agar-Lysine (LYS) plates (6.6% Oxoid lysine medium, 0.5% potassium lactate, 0.2% lactic acid). Plates were incubated at 28°C for 3 days. To identify the yeast present, approximately 25 colonies from each medium and each sampling point were picked randomly.

### Yeast Identification: RFLPs of the 5.8S-ITS rRNA Region and Sequencing of the D1/D2 Region from 26S rRNA Gene

Yeast isolates were identified by PCR-RFLP analysis of 5.8S-ITS rDNA according to Esteve-Zarzoso et al. (1999), using primers ITS1 and ITS4 (White et al., 1990). PCR products were digested without further purification by the restriction enzymes *Cfo*I, *Hae*III, *Dde*I, *Hinf*I, and *Mbo*I. The PCR products and their restriction fragments were separated by gel electrophoresis on 1.5% and 3% agarose gels, respectively. The sizes of the DNA fragments were estimated by comparison against a DNA ladder (100 bp Roche Diagnostics GmBh, Germany). The obtained restriction profiles were compared with previously reported profiles (Esteve-Zarzoso et al., 1999; Sipiczki, 2004; Baffi et al., 2010). One isolate was sent for sequencing of the D1/D2 domains of 26S rRNA was conducted using primers NL1 and NL4 to confirm yeast identification (Kurtzman and Robnett, 1998). The PCR products were purified and sequenced by Macrogen Inc. (Seoul, South Korea) using an ABI3730XL automated capillary DNA sequencer. The sequences were compared with those in GenBank and with those of the Type Strains using the BLASTN tool (NCBI). Identification at species level was achieved with homologies with type strains between 99.2% (*S. cerevisiae*) to 100% (*Hanseniaspora uvarum*). The sequences were deposited in the GeneBank NCBI database with the accession numbers KX272958 (*Aureobasidium pullulans*), KX272959 (*H. uvarum*), KX272960 (*Issatchenkia terricola*), KX272961 (*Lachancea thermotolerans*), KX272962 (*Starmerella bacillaris* synonim *Candida zemplinina*), KX272963 (*S. cerevisiae*), and KX272964 (*Saccharomycodes ludwigii*).

Biodiversity indexes were determined as in McDonald and Dimmick (2003).

### Yeast Typing

Isolates from *S. cerevisiae* were genetically characterized by the analysis of inter-delta regions, as described by Legras and Karst (2003) using the primers delta12 and delta21. *H. uvarum* and *C. zemplinina* isolates were typified according to Barquet et al. (2012) with two different combination of primers. Set A included primers 5CAG and TtRNASc while set B was composed of the primers ISSR-MB and TtRNASc. PCR products were separated by electrophoresis on 2% agarose gels. *H. uvarum* isolates were further characterized by RAPD-PCR using the M13 set of primers (Huey and Hall, 1989). The clustering was performed using the profiles obtained with the three sets of primers. The sizes of the DNA fragments were estimated by comparison against a DNA ladder (100 bp Roche Diagnostics GmBh, Germany).

### Chemical Analysis of Final Wines

pH values were determined by a pH meter MicropH2000 (Crison Instruments, Barcelona, Spain). Sugars (glucose and fructose), acetic acid, citric acid, malic acid, tartaric acid, and glycerol were quantified using the Miura one enzymatic autoanalyzer (BioGamma I.S.E. S.r.L., Rome, Italy) with corresponding enzymatic kits (BioSystems S.A., Barcelona, Spain).

## Results

### Fermentation Kinetics and Yeast Populations

**FIGURE 1:**
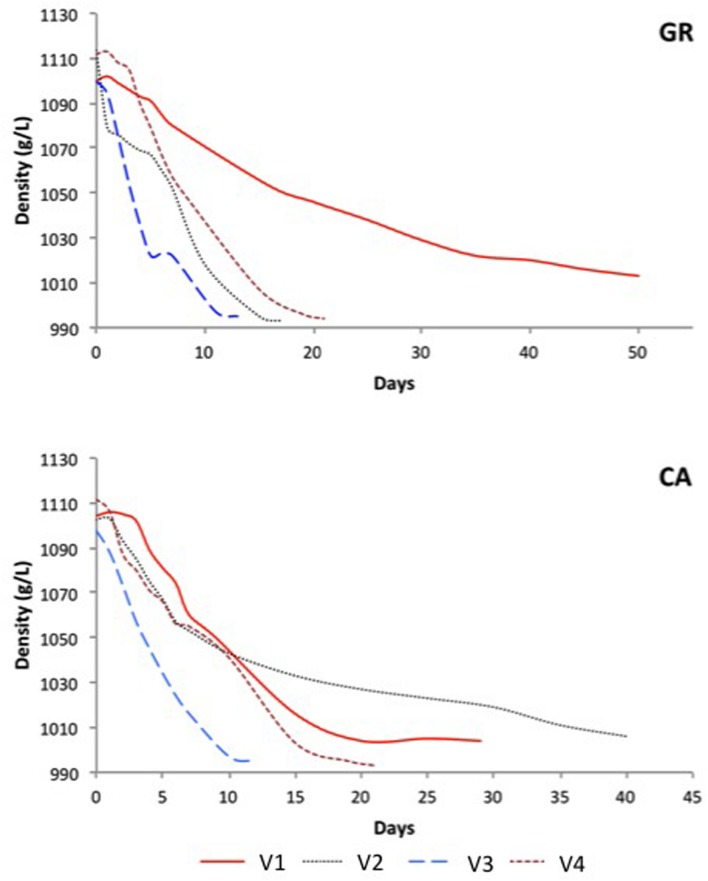
**Evolution of the different spontaneous fermentations.** GR: Grenache, CA: Carignan.

Fermentation processes measured by must density are represented in **Figure 1**. In all cases, the initial must density was between 1,098 and 1,114 g/L. The fermentation kinetics determined by density monitoring indicated that the eight spontaneous alcoholic fermentations observed progressed differently, as three experiments were complete after 10–15 days (V2-GR, V3-GR, and V3-CA), two after 20 days (V4-GR and V4-CA) and three fermentations were incomplete after 20 days (V1-GR, V1-CA, and V2-CA). Except in V2, a similar trend was observed in the fermentation kinetics of experiments performed with grapes from the same vineyard but of a different variety.

Yeast counts were registered at different sampling points when possible due to the growth of filamentous fungi, which hampered proper yeast visualization and isolation. The initial yeast counts ranged from 10^4^ to 10^6^ CFU/mL in both growth media. In all cases, typical growth kinetics were observed with high total yeast viability until the end of fermentation, with values of approximately 10^7^ CFU/mL. On the other hand, the growth of non-*Saccharomyces* species at this point was only observed in three fermentations, with values ranging from 10^5^ to 10^7^ CFU/mL. These species were present at the mid-fermentation point in all experiments, with counts between 10^6^ and 10^8^ CFU/mL.

### Yeast Identification and Population Dynamics

A total of 1,401 yeasts were isolated and identified from samples taken during spontaneous alcoholic fermentation. Eleven non-*Saccharomyces* species, as well as *S. cerevisiae*, were found. The most abundant yeast species was *H. uvarum*, followed by *S. cerevisiae*, *C. zemplinina* and *A. pullulans*. Smaller quantities of other species such as *C. intermedia*, *S. ludwigii* and *I. terricola* were isolated.

**FIGURE 2:**
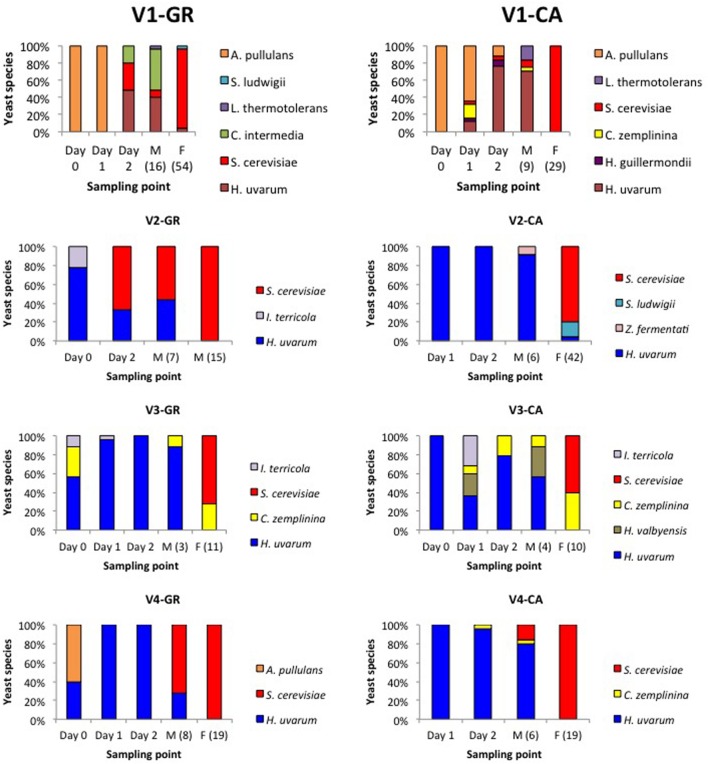
**Yeast population dynamics established by RFLP-ITS-PCR of YPD-cultured isolates**.

**Figure 2** shows the population dynamics during spontaneous vinifications of yeasts isolated in YPD medium. Obvious differences in species succession exist across the different experiments, influenced by the initial yeast load as well as by the endogenous vineyard microbiota. In the case of V4, only three species were identified in GR and CA, while the V1-GR and V1-CA fermentations were characterized by four common species and two species that were dependent on the grape variety. Between three and five different yeast species were involved in V2 and V3 fermentations.

**FIGURE 3:**
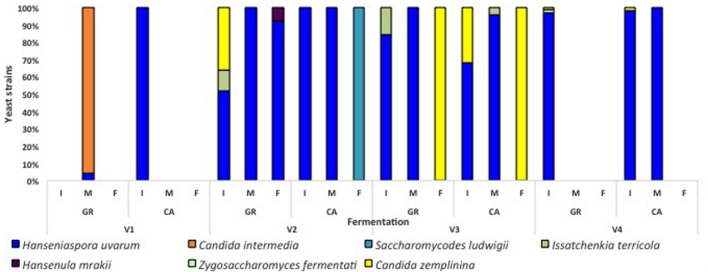
**Percentages of yeast isolates in lysine media along the different spontaneous fermentations.** *I* (initial), *M* (mid), and *F* (final) refer to the analyzed fermentation stages. Absence of bars means no isolates could be recovered from plates.

Globally, the first stages of fermentation (Days 0, 1, and 2) were characterized by the presence of several non-*Saccharomyces* species, particularly *H. uvarum*. In the case of V1 fermentations, *A. pullulans* represented more than 50% of the isolates found at the beginning of the process. *S. cerevisiae* was present during this initial phase in fermentations V1-GR, V1-CA, and V2-GR, while in other experiments this species appeared at the mid (V4-GR and V4-CA) or final points of fermentation (V2- CA, V3-GR, and V3-CA). The clear dominance of *S. cerevisiae* (60–100%) at later sampling points was observed in all fermentations. However, the coexistence of non-*Saccharomyces* species, particularly *H. uvarum*, *C. zemplinina*, and *S. ludwigii*, and the appearance of *S. cerevisiae* at the end of the fermentation process is noticeable in different experiments (V1-GR, V2-CA, V3-GR, and V3-CA). When plated in lysine medium (**Figure 3**), the species *Hansenula mrakii* was also found.

To estimate yeast biodiversity we calculated species biodiversity by species richness and the indexes of Shannon–Weiner and Simpson (**Table 1**). It can be seen that the first vineyard (the only one certified organic) had the highest biodiversity indexes, whereas the last one, the only fully conventional one has the lowest biodiversity.

**Table 1:** Biodiversity indexes in the studied vineyards.

	V1-GR	V1-CA	V2-GR	V2-CA	V3-GR	V3-CA	V4-GR	V4-CA
*S*	6	6	5	4	4	5	5	3
*H’*	1.41	1.45	1.03	0.93	0.94	1.14	0.84	0.54
*D*	0.74	0.70	0.57	0.52	0.50	0.59	0.47	0.30


**S*, Species richness; *H’*, Shannon–Weiner index; and *D*, Simpson index.*

### Yeast Typing

**FIGURE 4:**
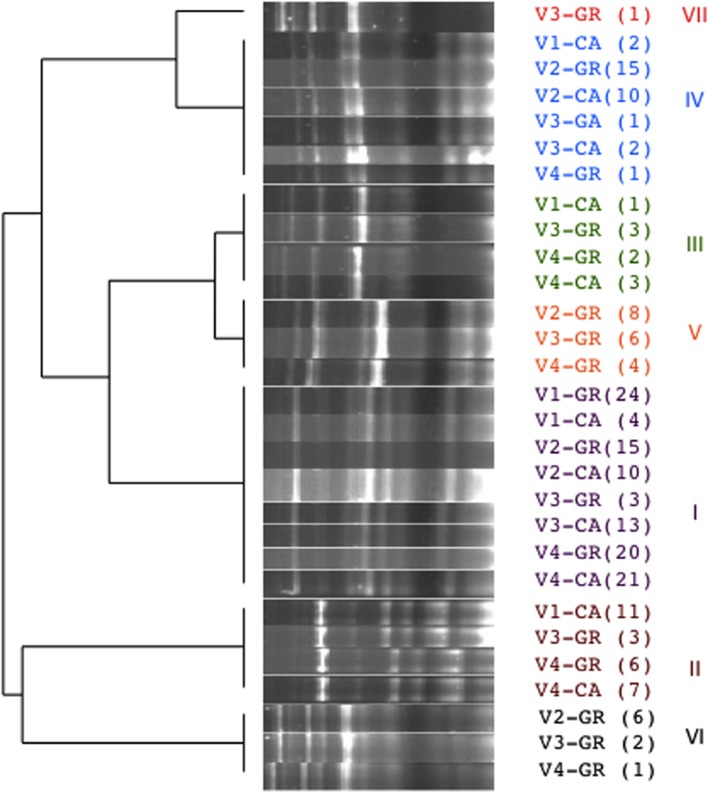
**Cluster analysis of the electrophoretic patterns of inter-delta PCR amplification obtained from representative isolates of *Saccharomyces cerevisiae* form each fermentation.** In brackets number of isolates with the same profile.

**FIGURE 5:**
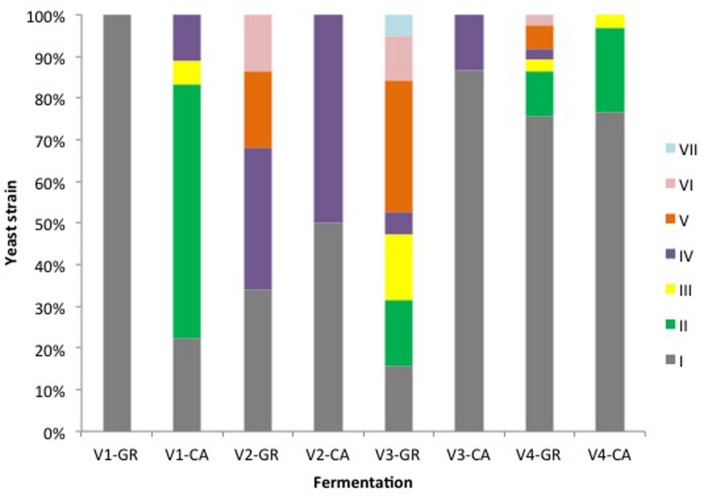
**Percentages of *S. cerevisiae* strains in different fermentations**.

A total of 315 isolates were typified from different species: *S. cerevisiae* (205), *H. uvarum* (98), and *C. zemplinina* (9). Seven electrophoretic patterns were observed in *S. cerevisiae* (**Figure 4**). **Table 2** and **Figure 5** show the distribution of the inter-delta profiles of *S. cerevisiae* isolates from the eight spontaneous fermentations studied. Some fermentations contained only one or two strains (V1-GR, V2-CA, and V3-CA), while others included all strains (V3-GR). In all vinifications, inter-delta profile I was present and was the predominant profile in most vinifications, while III, VI, and VII were isolated in smaller numbers. Profiles I, II, and IV were present in all the fermentative processes studied, while V and VI were not found during the initial stages and III and VII were only isolated at the final fermentation sampling points.

**Table 2:** Distribution of the different *Saccharomyces cerevisiae* strains among the fermenting grape musts and fermentation stages.

Profile	V1-GR			V1-CA			V2-GR			V2-CA			V3-GR			V3-CA			V4-GR			V4-CA			Σ
								
	*I*	*M*	*F*	*I*	*M*	*F*	*I*	*M*	*F*	*I*	*M*	*F*	*I*	*M*	*F*	*I*	*M*	*F*	*I*	*M*	*F*	*I*	*M*	*F*	
I	5	2	17	1	-	3	-	-	15	-	-	10	-	-	3	-	-	13	-	8	12	-	4	17	110
II	-	-	-	1	2	8	-	-	-	-	-	-	-	-	3	-	-	-	-	2	4	-	-	7	27
III	-	-	-	-	-	1	-	-	-	-	-	-	-	-	3	-	-	-	-	-	2	-	-	3	9
IV	-	-	-	-	-	2	6	3	3	-	-	10	-	-	1	-	-	2	-	-	1	-	-	-	31
V	-	-	-	-	-	-	-	5	3	-	-	-	-	-	6	-	-	-	-	-	4	-	-	-	18
VI	-	-	-	-	-	-	-	6	-	-	-	-	-	-	2	-	-	-	-	-	1	-	-	-	9
VII	-	-	-	-	-	-	-	-	-	-	-	-	-	-	1	-	-	-	-	-	-	-	-	-	1
Σ	24			18			44			20			19			15			34			31			205


**I*, grape must; *M*, mid fermentation; *F*, End of fermentation.*

**FIGURE 6:**
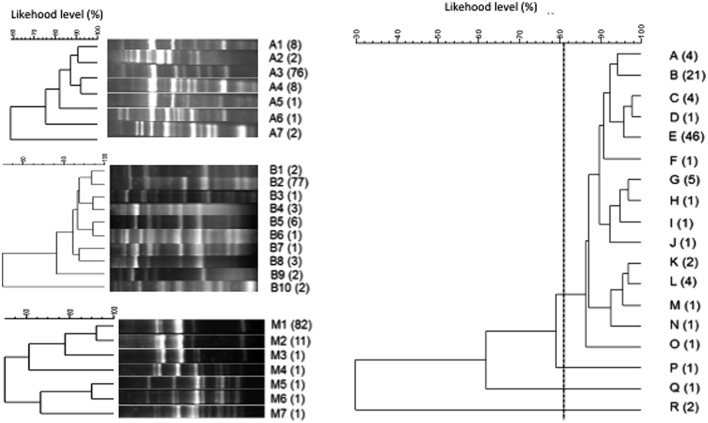
**Cluster analysis of the electrophoretic patterns obtained with three different sets of primers (RxA, RxB, and M13) of *Hanseniaspora uvarum* isolates.** The cluster obtained with the combination of the three analyses is also represented (right).

*Hanseniaspora uvarum* isolates in V2 fermentations were typified by combining the results obtained from primer sets A and B and M13 RAPD-PCR. As a result of the genetic characterization of *H. uvarum* combining the results of the tipification tests 18 different strains were differentiated (**Figure 6**). Each strain pattern was composed of between one and 46 isolates, and only one strain included isolates from two different sampling points (profile E).

*Candida zemplinina* isolates from fermentation V2-GR were studied, and six different combinations of profiles were obtained: four isolates corresponded to the same strain pattern, while the other five were classified individually as single strains.

### Chemical Analysis of Final Wines

The primary oenological parameters of the eight wines obtained are shown in **Table 3**. All wines contained less than 2 g/L of residual sugars and are thus considered dry. The only exception was V4-GR, which contained 4.46 g/L of residual sugars, which in laboratory scale fermentations is also often considered dry. The final pH values measured were between 2.92 and 3.45, and CA fermentations presented higher values than GR wines. Glycerol values ranged from 8.06 in V2-GR to 12.65 in V3-GR. The acidic contents were measured, and values close to 0.2 g/L were obtained for citric acid, while malic acid ranged from 0.53 to 2.16 g/L, the tartaric acid concentration varied from 0.3 to 2.48 g/L and acetic acid values were determined to be between 0.10 and 1.21 g/L. The quantification of ethanol was not consistent due to different lengths of fermentation and ethanol evaporation due to the small volumes involved. However, considering the low levels of residual sugars, no fermentations were stuck.

**Table 3:** Analytical parameters of final wines.

	Glucose + Fructose (g/L)	pH	Glycerol (g/L)	Malic acid (g/L)	Citric acid (g/L)	Tartaric acid (g/L)	Acetic acid (g/L)
V1-GR	0.56	2.92	10.09	1.20	0.26	2.48	1.20
V1-CA	0.02	3.40	10.91	2.16	0.34	0.88	0.80
V2-GR	1.12	2.99	8.06	0.67	0.20	1.34	0.77
V2-CA	0.10	2.99	10.99	0.53	0.13	2.41	1.21
V3-GR	0.06	3.22	12.65	0.67	0.12	0.78	0.97
V3-CA	0.07	3.31	11.09	0.87	0.22	0.01	0.71
V4-GR	4.46	2.98	9.90	0.80	0.17	1.50	0.30
V4-CA	0.29	3.45	12.49	0.91	0.13	0.30	0.10


## Discussion

In this work, the yeast population dynamics of eight different spontaneous vinifications of DOQ Priorat grapes were explored. Regarding fermentation kinetics, three different patterns were observed, as the fermentation lengths required for sugar consumption varied, from approximately 10–20 days and longer for sluggish fermentations.

Yeast isolates were identified by molecular techniques, and 11 non-*Saccharomyces* species as well as *S. cerevisiae* were found in the alcoholic fermentations, indicating that vineyards are an excellent source of yeast biodiversity. Although there are no enough number of vineyards analyzed, our results seems to indicate that organic handling could increase the biodiversity indexes, as observed by other authors (Setati et al., 2015). All yeast species isolated in this study have been previously described in grapes or in wine related environments (Renouf et al., 2005; Barata et al., 2012; Ortiz et al., 2013; Alessandria et al., 2014; Jolly et al., 2014). The main non-*Saccharomyces* yeast species isolated belong to the genera *Hansenianspora* and *Candida*, which have been commonly associated with grape juice and are gradually replaced by *S. cerevisiae* during alcoholic fermentation (Fleet, 2003; Ocón et al., 2010). In this sense, yeast population dynamics along the eight DOQ Priorat spontaneous fermentations was examined. A typical species succession trend was observed in all fermentations, although differences among the main non-*Saccharomyces* species were noticeable, such as the presence of *A. pullulans* in some of the experiments. This black yeast-like fungus is a common inhabitant on the surface of healthy grapes, which would explain its presence at the beginning of the fermentation process (Fleet, 2003; Sun et al., 2014). The only previous study performed in Priorat (Torija et al., 2001) studied the yeast population dynamics of GA fermentation over a period of three years, and determined that the main non-*Saccharomyces* species isolated was *C. stellata* (later known as *C. zemplinina* or *S. bacillaris*). An ecological analysis of yeast compositions conducted during six different years on different grape musts from nearby vineyards (although outside the DOQ Priorat) revealed that *H. uvarum* or *C. stellata* dominated the first stages of fermentation, depending on the experiment (Beltran et al., 2002). In the present study, *H. uvarum* was, excepting the cases where *A. pullulans* predominated, the dominant non-*Saccharomyces* species, as has been reported by other authors (Querol et al., 1990; Constanti et al., 1997; Bezerra-Bussoli et al., 2013).

In addition to being abundantly present during the beginning of spontaneous alcoholic fermentations, *H. uvarum* and *C. zemplinina* are considered interesting yeast species both for inclusion in starter cultures that aim to emulate natural fermentation, as well as from an aromatic point of view, as both yeast species are likely to affect the sensory properties of the final wine (Fleet, 2008; Jolly et al., 2014) . However, the production of volatile compounds and other molecules related to oenological parameters has proven to be strain dependent (Romano, 2003; Comitini et al., 2011; Loira et al., 2014), which highlights the relevance of conducting a strain characterization and selection process to obtain a desired outcome. Ecological studies generate large microbial collections that need to be genetically characterized to differentiate strains, simplify phenotypical characterization and provide a better conception of the winemaking process.

In the present study, *S. cerevisiae* isolates were typified by delta-elements PCR resulting seven different electrophoretic profiles from eight spontaneous fermentations. More than one strain was found in each experiment, indicating the coexistence of several strains during the vinification process, as has been specifically indicated in the same area (Torija et al., 2001) or widely reported (Fleet, 1993; Tofalo et al., 2013). These data support the idea of designing starter cultures that include more than one native strain of *S. cerevisiae* to mimic spontaneous fermentations. In fact, the practical application of this study has been the development of mixed inoculum containing the three main strains of *S. cerevisiae* observed in the present study (strains I, II, and IV). Additionally, some strains were exclusively found in one grape variety, even when harvested in different vineyards, which highlights the relationship between microbial diversity and varietal character. The absence of *S. cerevisiae* at the beginning of the grape must fermentation is well-known in culture-dependent studies, due to its near absence in grapes (Fleet, 1993); although in some cases it has been found when the sanitary status of the grapes was unusual (Beltran et al., 2002). However, its capacity to lead the fermentation process and interact with other non-*Saccharomyces* species leads to the recovery of only *S. cerevisiae* at the end of fermentation (Fleet, 1993).

The two main non-*Saccharomyces* species found, *H. uvarum* and *C. zemplinina* isolates from V2 spontaneous fermentations, were also typified. Both species included abundant strain patterns, although one main profile was found, and all *H. uvarum* strains were grape variety dependent. The biodiversity found among non-*Saccharomyces* isolates was much greater when compared with *S. cerevisiae*, as only four *S. cerevisiae* strains were found in V2 fermentations. The combination of different typing methods can result in very different results. In fact, using only one of the methods the profile diversity could be much lower than that from the combination of several methods. Some authors that applied combined analysis in *Saccharomyces*, already observed this fact (Fernández-Espinar et al., 2001; Schuller et al., 2004). However, the methods for Non-*Saccharomyces* analysis are still far from being standardized and thus, comparative studies have been recently reported (Masneuf-Pomarede et al., 2015; Albertin et al., 2016). In our hands, the combination of the three analyses has provided much higher polymorphism increasing from 7 or 10 different profiles to 18 profiles after the combination of different methods. Thus, we consider that so far the use of a single method for typing non-*Saccharomyces* is not conclusive enough.

In addition to the different molecular typing methods used, this difference may be due to the high populations of non-*Saccharomyces* species found at the beginning of alcoholic fermentation. Most ecological studies based on the microbial description of spontaneous fermentation are focused on the analysis of *S. cerevisiae* populations; therefore, the genetic typing of non-*Saccharomyces* isolates is often unexplored. One exception is a study published by Capece et al. (2011), based on the characterization, in wines, of non-*Saccharomyces* SO_2_ tolerant yeasts by RAPD fingerprinting, with the aim of constructing a collection of wild strains capable of maintaining the specific sensory characteristics of Inzolia wine.

## Conclusion

This study provides a testimony for the remarkable yeast species and strain heterogeneity associated with alcoholic fermentations carried out by the wild yeasts naturally present in four different DOQ Priorat vineyards and in two different red grape varieties: GA and CA. This yeast community is likely to leave a footprint in the final wines, which will be part of the distinctive characteristic of the wines of a given region. The defense of a given area typicality often leads to the use of spontaneous fermentations which may produce uncontrolled fermentations with unwanted and deleterious effects. A multi-strain and multi-species starter with selected yeast of the available and more characteristic strains and species from a given region can provide the typicality of that region without the inconvenience of the uncontrolled fermentation. Thus, the description of this microbial diversity can be the first step of the selection of a consortium of native yeast microbiota emulating spontaneous fermentation that could be used for the production of wines exhibiting the Priorat footprint.

## Author Contributions

BP: Design experiments, perform experiments, analyze results, result discussion, and writing the manuscript. DG-F, BG, and IP: Perform experiments and analyze results. BE-Z and GB: design experiments, analyze results, and result discussion. AM: design experiments, analyze results, result discussion, writing the manuscript, and rise funding.

## Conflict of Interest Statement

The authors declare that the research was conducted in the absence of any commercial or financial relationships that could be construed as a potential conflict of interest.
